# Primary Tuberculosis of Hand Soft Tissue 

**Published:** 2016-09

**Authors:** Mohd Altaf Mir, Imran Ahmad, Mihd Yaseen

**Affiliations:** Department of Burns, Plastic and Reconstructive Surgery, Jawaharlal Nehru Medical College, AMU, Aligarh, India

**Keywords:** Tuberculosis, Flexor tendon sheath, Cutaneous, Hand

## Abstract

The musculoskeletal extrapulmonary tuberculosis is uncommon, the upper extremity tuberculosis is not common and the mycobacterial involvement of skin of hands and synovial sheath of tendons is rare. This prospective observational study was undertaken between August 2014 and December 2015 in a tertiary referral hospital. Patients attending with suspected primary tuberculosis of soft tissue of the hand were included. Anteroposterior and lateral radiographs of the hand, wrist and lung and MRI were undertaken. A diagnosis of tuberculosis was made based on histology, and positive culture for *Mycobacterium*. Split thickness skin grafting was done after excision of tubercular cutaneous ulcers of hand. Postoperatively patients were treated with home based anti-tubercular chemotherapy. Lesions of synovial sheath of tendons were excised. Splints or plaster slabs were used in all patients. There were 3 males and 2 females with a mean age of 44.75±6.61 years (19-48 years). Based on clinical suspicion, plain radiographs and MRI, there were 3 patients with involvement of synovial sheath of tendons and 2 patients with involvement of skin of hand. Tuberculosis was confirmed histologically and *Mycobacterium bovis* was confirmed microbiologically. The delayed diagnosis is often due to slow progression and numerous differential diagnoses, which often leads to complications. Early radical excision of the infected tissues combined with anti- tubercular multidrug therapy gives good functional results and prevents recurrence.

## INTRODUCTION

The musculoskeletal extrapulmonary tuberculosis is uncommon accounting for about 10-15% of all extrapulmonary tuberculosis.^1^ The upper extremity tuberculosis is not common^2^ and the mycobacterial involvement of skin of hands and synovial sheath of tendons is rare. *Mycobacterium bovis* infection accounts for 5-10% of tuberculosis.^2^ The source of *Mycobacterium bovis* for humans is the use of unpasteurized milk, direct contact with infected animals like cow and goat, and inhalation of contagious aerosols.^3^ The infection may be also acquired by workers at slaughter houses.^3^ Synovial sheath tuberculosis may present with chronic synovitis with or without swelling. The condition may mimic rheumatoid arthritis, sarcoidosis or soft tissue sarcoma. The diagnosis of tuberculosis of synovial sheath of tendon is usually achieved with magnetic resonance imaging and confirmed by histopathology and tubercular cultures. However, the diagnosis of tubercular skin lesions of hand are achieved clinically and confirmed by histopathology and tubercular cultures.^3^ Here, we described cases of primary tuberculosis of soft tissue of hand.

## CASE REPORT

This was a prospective observational study undertaken between August 2014 and December 2015 in a tertiary referral hospital. Patients attending with suspected tuberculosis of soft tissue of the hand were included. Those with a past history of operative treatment were excluded. There were 3 males and 2 females with a mean age of 44.75±6.61 years (19 to 48 years). A detailed history and examination were recorded. A full blood count, ESR, and HIV, hepatitis B and C testing were performed following informed consent. Anteroposterior and lateral radiographs of the hand and wrist were taken in all cases. 

MRI was undertaken in patients with suspected tubercular soft tissue swelling in the hand. All patients underwent chest radiography to exclude healed or active pulmonary tuberculosis. A diagnosis of tuberculosis was made on the basis of characteristic histopathology, a positive culture for *Mycobacterium* after excision of the lesion. Split thickness skin grafting (STSG) was done after excision of tubercular cutaneous ulcers in both the cases. Postoperatively patients were treated with home based anti-tubercular chemotherapy (ATT). Splints or plaster slabs were used for a short time in all patients after operative management except in case 1 who required tendon repair and splintage was continued for 6 weeks. All patients were followed up regularly. We represent here two cases of tuberculosis of synovial sheath of tendon and one case of cutaneous tuberculosis of hand.

*CASE 1*

**Fig. 1:**
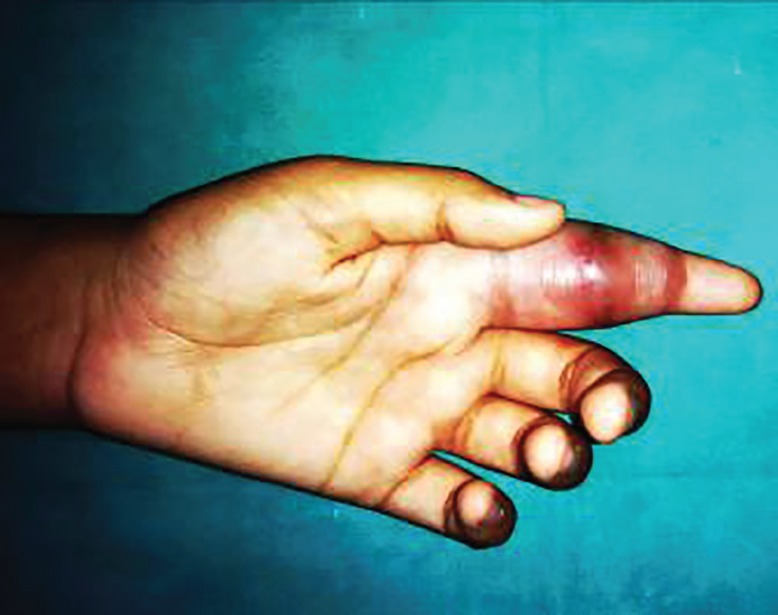
Preoperative photograph

**Fig. 2:**
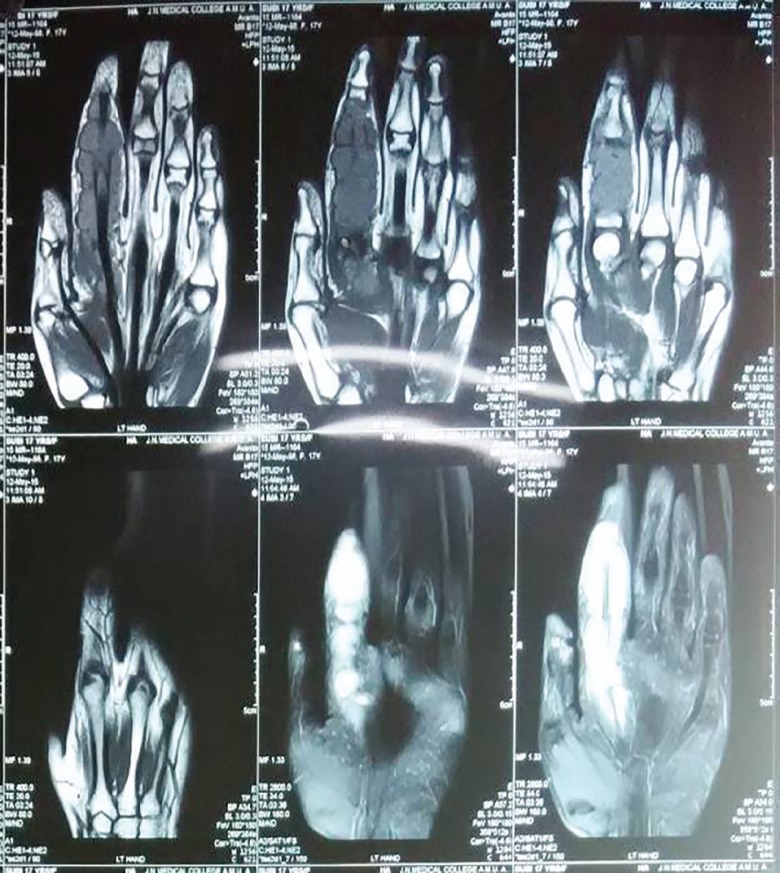
Magnetic resonance image

**Fig. 3:**
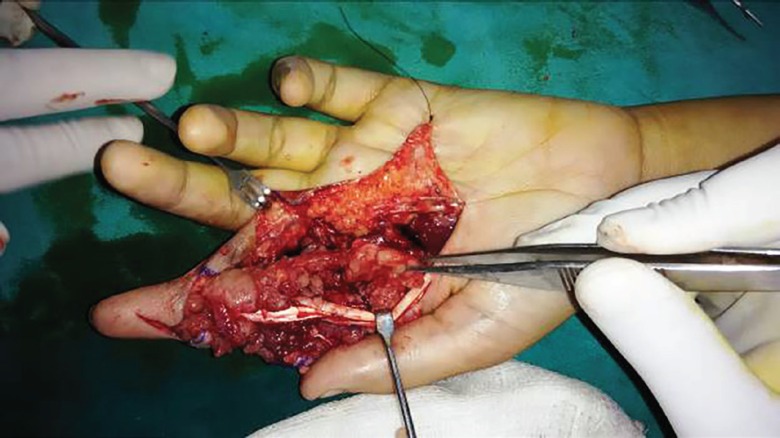
Intraoperative photograph showing lesion

**Fig. 4:**
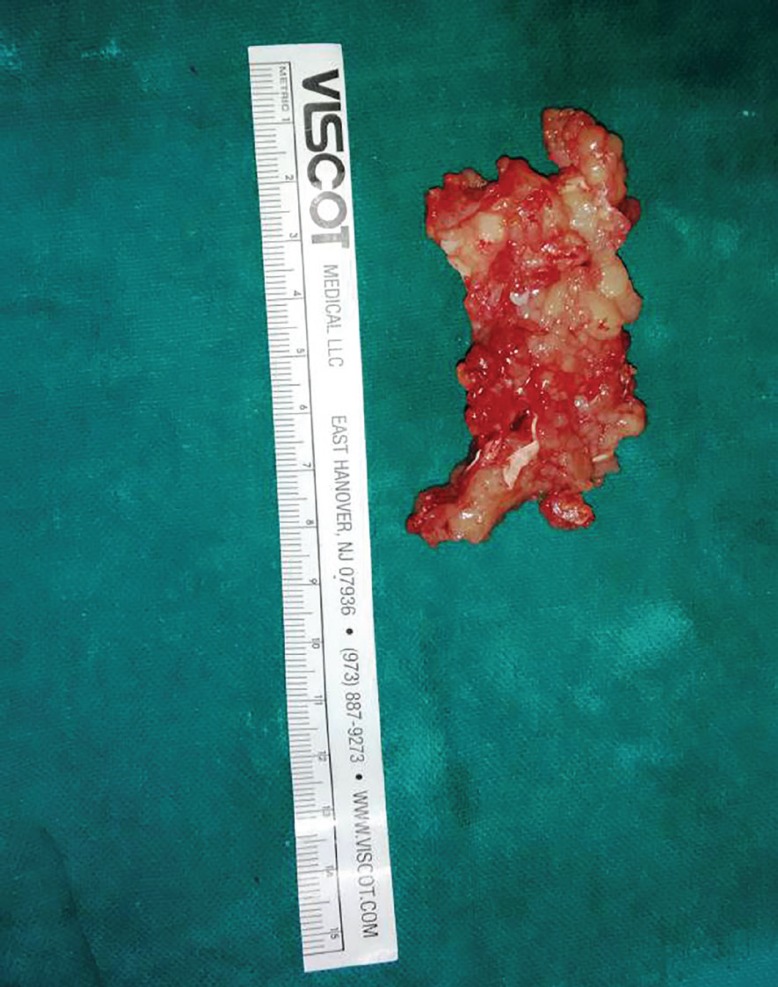
Excised specimen

A 19 year old female presented with painless swelling Zone II index finger of left hand of 2 years duration restricting the flexion of index finger at inter-phalygeal and metacarpo-phalyngeal joints (Figure 1). She has sustained an injury to the same finger with the knife 3 years back. A radiograph of the left hand shows soft tissue swelling zone II left index finger without any bony lesion. Magnetic resonance image of left hand shows contrast enhanced soft tissue lesion arising from flexor tendon sheath zone II of left index finger (Figure 2). On surgical exploration of flexor compartment of left index finger zone II with Brunner’s incision, the swelling arising from flexor tendon sheath of flexor digitorum profundus (FDP) and superficialis (FDS) tendons fixed at the chiasma of FDS (Figure 3), has been excised and the specimen (Figure 4) send for histopathological and microbiological examination. 

**Fig. 5:**
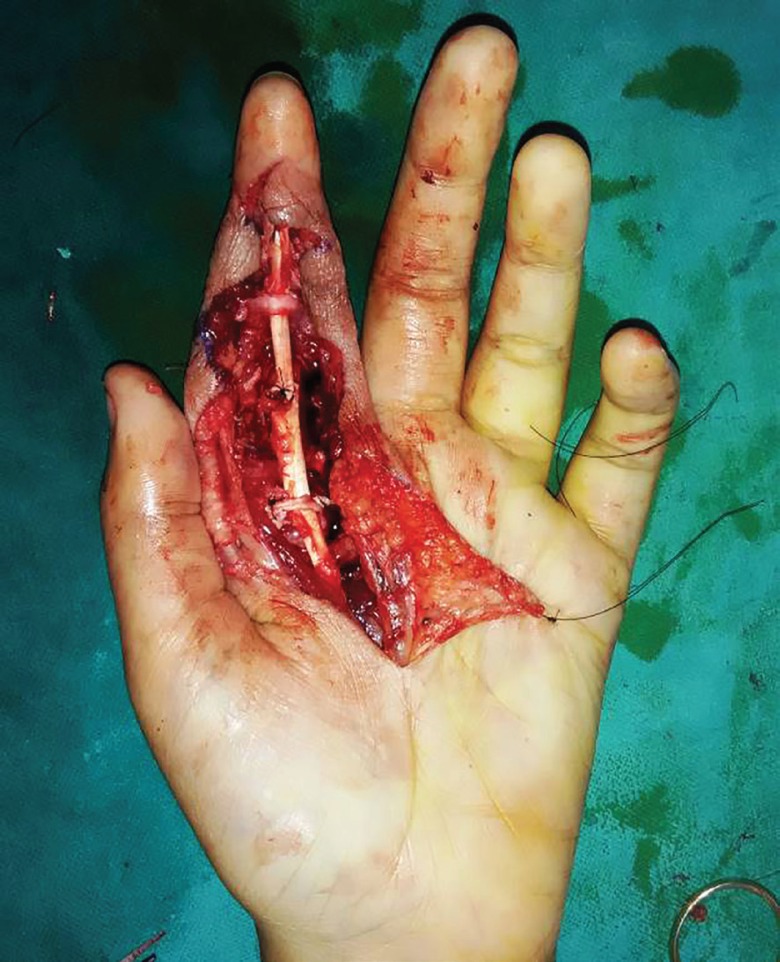
Intraoperative photograph after excision of lesion

**Fig. 6:**
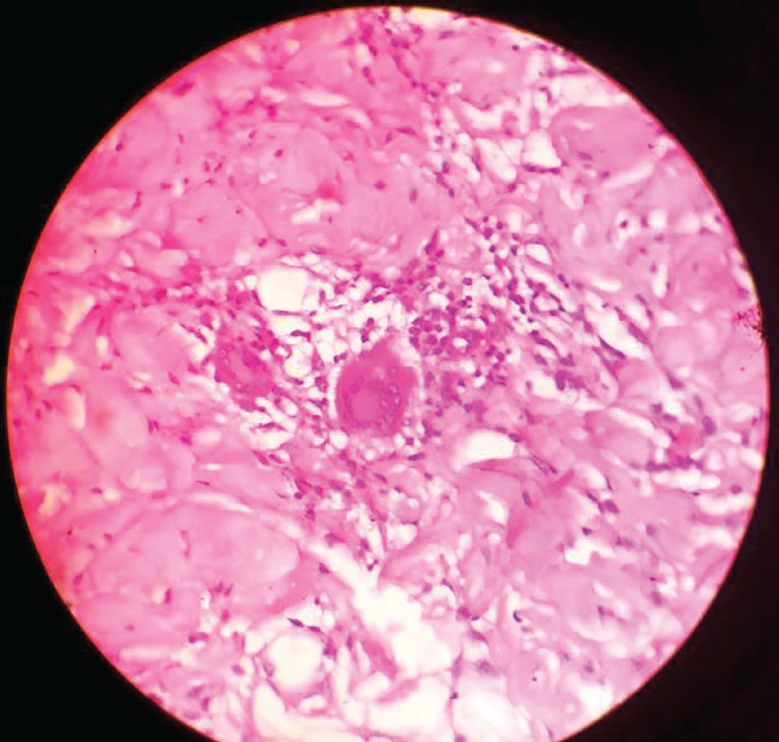
Histopathological microphotograph

The flexor tendons and pulley system of zone II left index finger has been repaired as is shown in (Figure 5), and Brunner’s incision closed. Histopathological examination of the excised specimen confirmed the diagnosis of granulomatous tuberculosis as evidenced by rice bodies (Figure 6). The aerobic and anaerobic cultures were negative. The tubercular culture using BACTEC confirmed the presence of *M. bovis*. There is satisfactory functional recovery of hand (Vedio1) and she has completed anti-tubercular therapy under the national tuberculosis control program of our country.

*CASE 2*

**Fig. 7:**
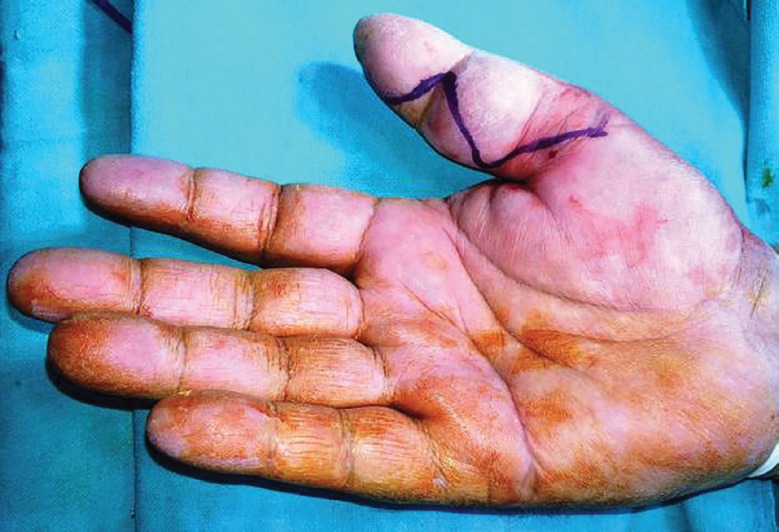
Preoperative photograph

**Fig. 8:**
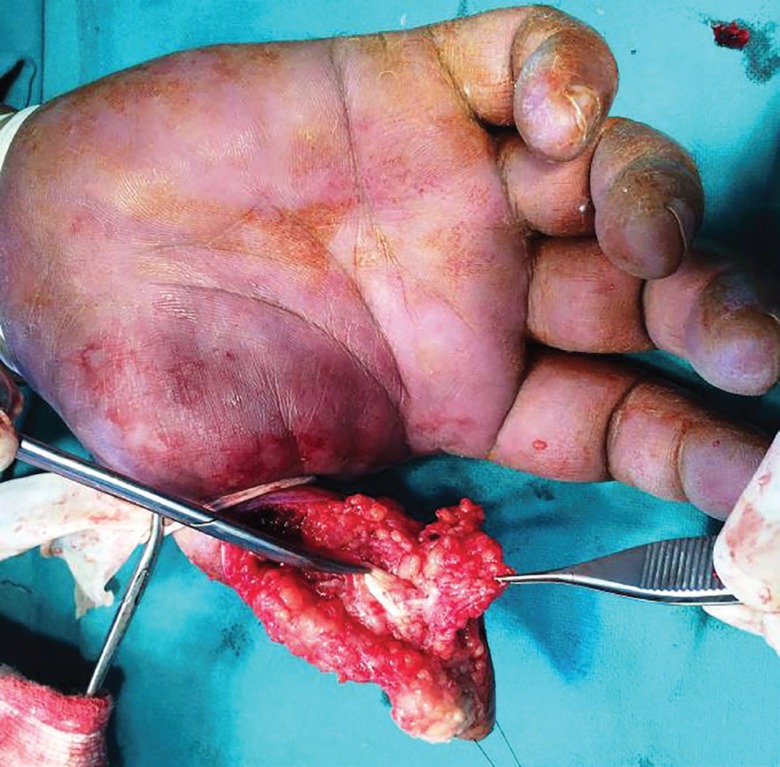
Intraoperative photograph

**Fig. 9:**
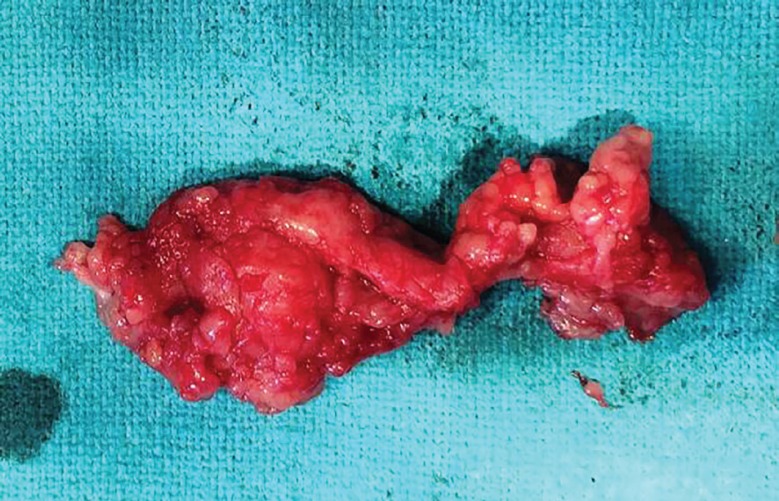
Photograph of excised specimen

A 45 year old male chef presented with painless swelling Zone II of thumb of right hand of 3 years duration restricting the flexion of thumb at inter-phalyngeal joint (Figure 7). He has sustained an injury to the thumb 5 years back. A radiograph of the right hand shows soft tissue swelling zone II right thumb without any bony lesion. Magnetic resonance image of right hand shows contrast enhanced soft tissue lesion arising from flexor tendon sheath zone II of right thumb. On surgical exploration of flexor compartment of right thumb with Brunner’s incision, the swelling arising from flexor tendon sheath of (FPL) flexor policis longus (Figure 8), has been excised and the specimen (Figure 9) send for histological and microbiological examination.

**Fig. 10:**
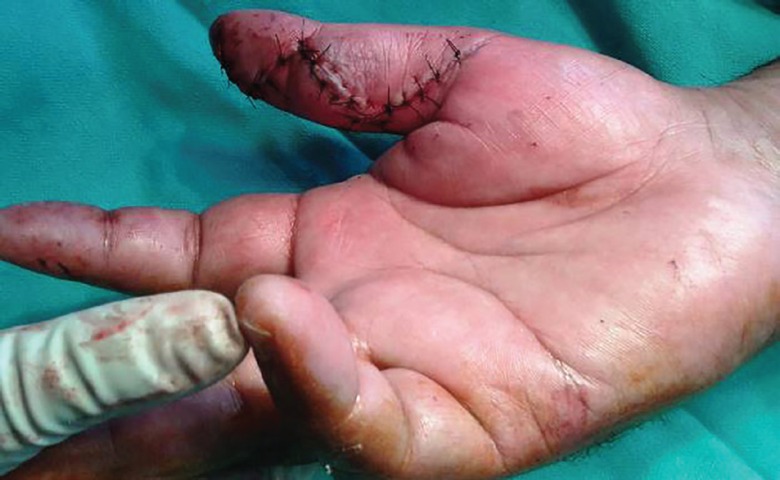
Postoperative photograph

The FPL was intact as shown in (Figure 8), and Brunner’s incision closed. The wound healing was satisfactory (Figure 10). Histological examination of the excised specimen confirmed the diagnosis of granulomatous tuberculosis as evidenced by rice bodies. The aerobic and anaerobic cultures were negative. The tubercular culture using BACTEC confirmed the presence of *M. bovis*. There is satisfactory functional recovery of hand and he has completed anti-tubercular therapy under the national tuberculosis control program of our country.

*CASE 3*

**Fig. 11:**
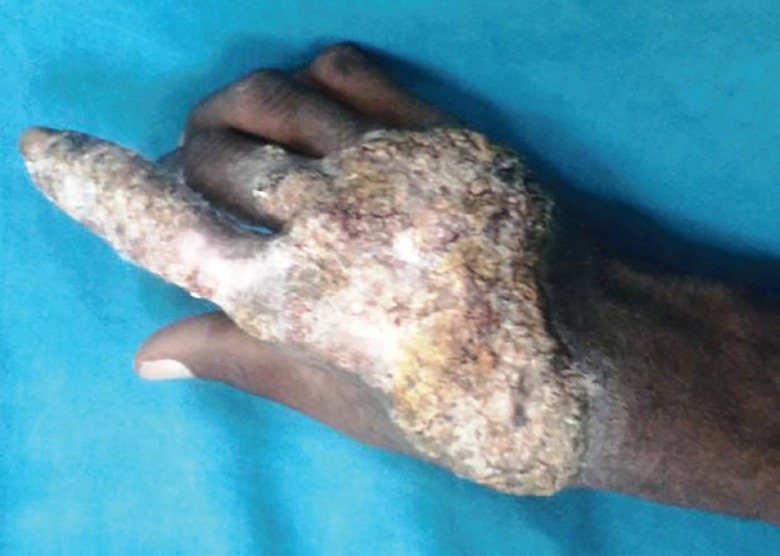
Preoperative photograph

**Fig. 12:**
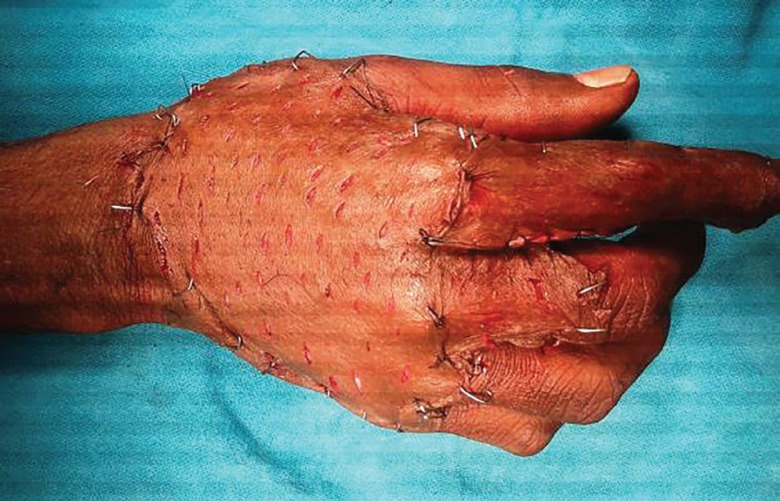
Intraoperative photograph after excision and Split thickness skin grafting

**Fig. 13:**
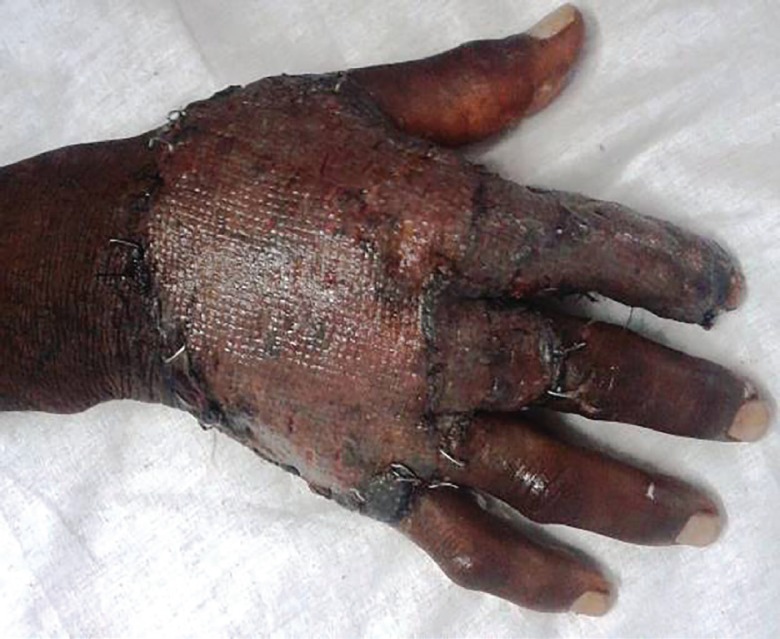
Postoperative photograph on first dressing

**Fig. 14:**
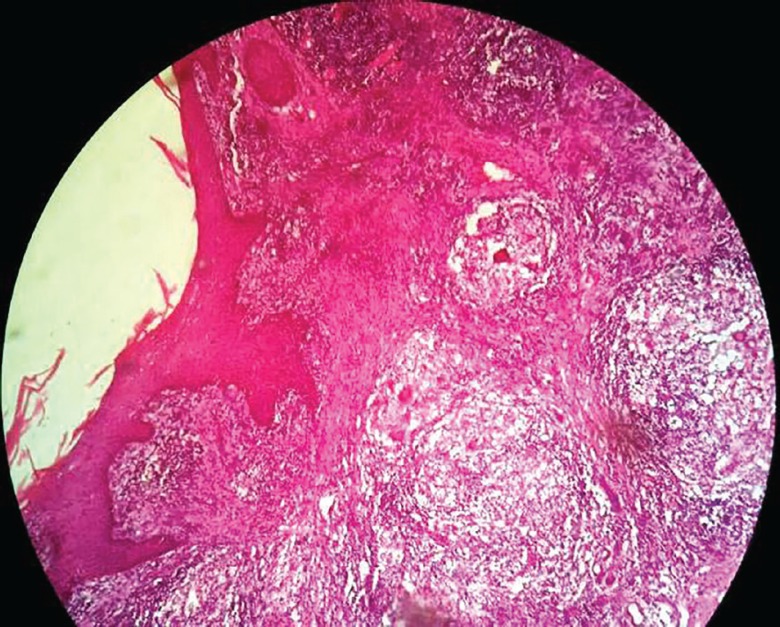
Histopathological microphotograph

A 48 year old male milkman presented with progressive ulcerative lesion dorsum of right hand since 4 years (Figure 11). A radiograph of the right hand excluded any bony lesion. Magnetic resonance image of right hand excluded any tendon sheath lesion. The lesion has been excised and the specimen sent for histopathological and microbiological examination. The postoperative wound was grafted with STSG (Figure 12). The grafted site was inspected for graft taken on 5^th^ day (Figure 13). Histopathological examination of the excised specimen confirmed the diagnosis of granulomatous tuberculosis (Figure 14). The aerobic and anaerobic cultures were negative. The tubercular culture using BACTEC confirmed the presence of *M. bovis*. There is satisfactory functional recovery of hand and he has completed anti-tubercular therapy under the national tuberculosis control program of our country.

The patients with suspected primary tuberculosis of soft tissue of hand were referred to Hand clinic of the department of Plastic and Reconstructive Surgery. There were 3 males and 2 females with a mean age of 44.75±6.61 years (19 to 48 years). Based on clinical suspicion, plain radiographs and MRI, there were 3 patients with involvement of synovial sheath of tendons and 2 patients with involvement of skin of hand. Tuberculosis was confirmed in all the cases by histopathological examination. *M. bovis* was confirmed microbiologically in all the cases as etiological agent.

## DISCUSSION

Extrapulmonary tuberculosis of flexor tendon sheath of hand may occur after hematogenous spread of *Mycobacterium* from lungs, lymphatic tissue and other viscera.^2^^,^^4^ The *M. bovis *infection may occur due the use of unpasteurized milk, direct contact with infected animals like cow and goat, inhalation of contagious aerosols, and is more common in slaughter house workers.^3^ Flexor tendon sheath tuberculosis of hand significantly reduce mobility of joints.^2^^,^^5^^-^^7^ and also cause compression of nerves.^2^^,^^5^^,^^7^ Tendon ruptures has been also reported in the literature.^8^^,^^9^ Magnetic resonance imaging findings may vary with severity and progression of the disease. It may show thickening of the synovial membrane with increased vascularization, fluid within the tendon sheath, reactive inflammation around the tendon, or swelling of the tendon sheaths.^10^^-^^12^ Bacteriology of the tissue culture specimen is often required to detect the bacterial infection.^13^

There are three histological forms of tuberculosis of flexor tendon sheath as a result of the progression of the disease, the resistance of the individual, and the varying virulence of the microorganism. In the first stage, the tendon is replaced by vascular granulation tissue. In second stage, the sheath is obliterated by fibrous tissue, fluid is confined within the sheath and rice bodies may appear due to caseation. In third stage, the tendon may be replaced with strands of tissue and may rupture spontaneously. When healing by fibrous tissue formation fails to curtail the pathologic process, extensive caseation and granulation occur which may lead to sinus formation and superimposed secondary infection.^8^^,^^9^^,^^14^^,^^15^

Rice bodies represent fibrinous masses (tubercles) which are present in 50% of cases.^15^^,^^16^ In tuberculosis early diagnosis and radical surgical debridement and anti-tubercular therapy is essential for management.^13^^,^^17^^,^^18^ Flexor and Extensor tendon involvement and ruptures may need repair or tendon grafting with reconstruction of pulley system. The term “lupus” to describe an ulcerative skin disease dates to the late thirteenth century, though it was not until the mid nineteenth that two specific skin diseases were classified as Lupus erythematosus and Lupus vulgaris. The diagnosis of cutaneous tuberculosis is complicated and requires a full workup, including a detailed history and physical examination; careful consideration of clinical presentation Mycobacterial culture remains the most reliable method to determine the presence of mycobacteria.^19^

In our study lesions of synovial sheath of tendons of hand, MRI shows contrast enhancement of lesions, histopathological examination of all excised specimen is suggestive of tuberculosis and microbiological examination of all specimen confirmed *M. bovis* infection. Primary tuberculosis of soft tissue of the hand is rare. The delayed diagnosis is often due to slow progression and numerous differential diagnoses, which often leads to complications. Early radical excision of the infected tissues combined with anti- tubercular multidrug therapy gives good functional results and prevents recurrence.

## CONFLICT OF INTEREST

The authors declare no conflict of interest.
